# Longitudinal Deformation of Deep Shield Tunnels Caused by Upper Load Reduction

**DOI:** 10.3390/ma14133629

**Published:** 2021-06-29

**Authors:** Jinlei Zheng, Shaohui He, Yiming Li, Jiaxin He, Jihua He

**Affiliations:** 1 School of Civil Engineering, Beijing Jiaotong University, Beijing 100083, China; zhengjinlei@yeah.net (J.Z.); 19115028@bjtu.edu.cn (Y.L.); 20115019@bjtu.edu.cn (J.H.); 2 China Railway 23 Construction Bureau, Ltd. the Sixth Group, Chongqing 400800, China; hubeihjh@126.com

**Keywords:** deep shield tunnel, foundation beam, upper load reduction, longitudinal deformation

## Abstract

Above-crossing excavations may cause uplift damages on existing shield tunnels. Therefore, to accurately calculate the deformation of shield tunnels is very necessary for geotechnical engineers. At present, the single-sided elastic foundation beam model is usually used in longitudinal deformation calculations for shield tunnels, which overestimates the uplift of deep shield tunnels. Because of the existence of the ground arch, deep shield tunnels are subjected to two-sided foundation reaction forces. Therefore, this paper proposes a partial missing double-sided elastic foundation beam model and the related fourth-order partial differential equations. In this model, the shield tunnel is subjected to double Winkler foundation springs and is simply considered a Euler–Bernoulli beam. A two-stage analysis method is used to solve the problem. First, the vertical unloading stress due to the above-crossing tunnelling at the tunnel location is calculated through Mindlin’s solution. Second, the deformation response of the beam subjected to an unloading stress is calculated by the finite difference method. Two engineering cases are used to verify the research. The results indicate that the proposed model is more accurate than traditional models in predicting the maximum uplift value, which is basically consistent with the observations. Due to the existence of segment staggering, the longitudinal influence range of the calculation by two models is larger than the actual measurement.

## 1. Introduction

In recent years, the overburden depth of subway tunnels has rapidly increased. As a result, various projects involving above-crossing tunnelling are becoming increasingly common. During the construction of tunnelling, the ground stress is released due to the excavation of the stratum, and the balanced ground stress field is broken, which will inevitably induce adverse effects, such as upward deformation and segment stagger, for the existing tunnels. Compared to mine tunnels, a shield tunnel has a weaker longitudinal deformation resistance because there are segmental lining joints. With increasing subway running speed, the deformation requirements of subway tunnels are becoming increasingly demanding. Therefore, serious concerns have been increasingly raised about the deformation response of underlying subway tunnels, especially existing shield tunnels, which are the focus of this study, due to the upper load reduction.

At present, scholars have studied the effects of adjacent construction on existing tunnels through various approaches such as field observations, numerical analyses, and theoretical derivations, and have obtained useful conclusions. Based on field observations, He et al. [1], Simpson et al. [2], Shi et al. [3], Chen et al. [4] and Chen et al. [5] obtained the deformation laws of existing tunnels during adjacent tunnelling progresses and provided control recommendations. However, the observed deformation laws notably vary due to engineering designs, stratum environments, construction modes and other factors. As a consequence, field experiences cannot accurately predict the deformation values of the existing tunnels in similar projects. Numerical analysis methods can consider the interaction between underground structures and surrounding soils and simulate the entire construction process. Therefore, it is mainly used to optimize the construction schemes for adjacent excavation [6,7,8,9,10,11]. However, to obtain the ideal calculation results, much modelling, calculation work and professional simulation software is required, which limits the application of the numerical analysis. Therefore, more widely applicable theoretical calculation methods to evaluate the effects on and risks to existing shield tunnels are necessary.

Among the current theoretical calculation methods, the elastic foundation beam model is most widely used. Its basic principle is to simplify the shield tunnel into an infinitely continuous beam on an elastic foundation. The main difference in these studies is the combination of different foundation models and types of beams. Zhang et al. [12] and Liang et al. [13] selected a Winkler foundation that only considered the interaction between tunnel structures and surrounding soils, where the foundation springs are independent of each other. They simplified the shield tunnel as a Euler–Bernoulli beam that only accounted for flexural deformation under bending. Considering the stratigraphic continuity, Zhang et al. [14] used the Pasternak two-parameter foundation model. Wu et al. [15] considered that the shearing dislocation between tunnel rings should not be ignored and selected a Timoshenko beam with the equivalent bending stiffness and shear stiffness. Based on the predecessors’ researches, Liang et al. [16] and Wu et al. [17] used a two-parameter foundation model and a Timoshenko beam together to predict the longitudinal deformation of shield tunnels. Liu et al. [18] further improved the foundation beam model, in which the tunnel is represented by a series of short beams resting on a Winkler foundation connected by tensile springs, compressional springs and shear springs. For some special conditions, Cheng et al. [19] employed a Timoshenko beam resting on the Winkler foundation to evaluate the deformation of shield tunnels with multiple discontinuities in strata. Through the continuous improvement of the foundation beam model, the prediction results are increasingly close to the results from engineering practice.

However, by analysing the forces, it can be found that the beams in the aforementioned models are only subjected to single-sided foundation reaction forces under shield tunnels. Moreover, the calculation results are similar to the observations when the shield tunnels are shallow, but the calculation obviously overestimates the deformation of deep shield tunnels. For example, Zhang et al. [12] used a model based on a Euler–Bernoulli beam on a Winkler foundation to calculate the deformation of an existing shield tunnel adjacent to a new foundation pit in Shanghai, China. The calculated maximum upheaval was 48% larger than the measured maximum upheaval. Liang et al. [13] calculated the vertical displacements of existing twin tunnels due to the above-crossing tunnelling in Shanghai and found that the calculated maximum values were 60% and 87% larger than the measured results.

Aiming at the shortcomings of previous studies on the longitudinal deformation of deep shield tunnels due to the above-crossing construction, a simplified calculation model based on the basic Winkler foundation and Euler-Bernoulli beam is established, which can be called the partial missing double-sided elastic foundation beam model. The author carefully derives a fourth-order partial differential equation with coefficient variables and provides a scheme to solve the equations suing the finite difference method. Finally, the model is verified by two measured engineering cases.

## 2. Model Analysis

### 2.1. Longitudinal Deformation Mechanism of Shield Tunnel

The above-crossing tunnelling will break the balanced ground stress field and cause the heave and additional bending moment of the underlying tunnel. If the deformation is too large, the safety of the tunnel structures and running trains will be seriously threatened. According to the different buried depth, shield tunnels can be divided into shallow tunnels and deep tunnels. These two different types of tunnels have different mechanical characteristics.

**Figure 1:**
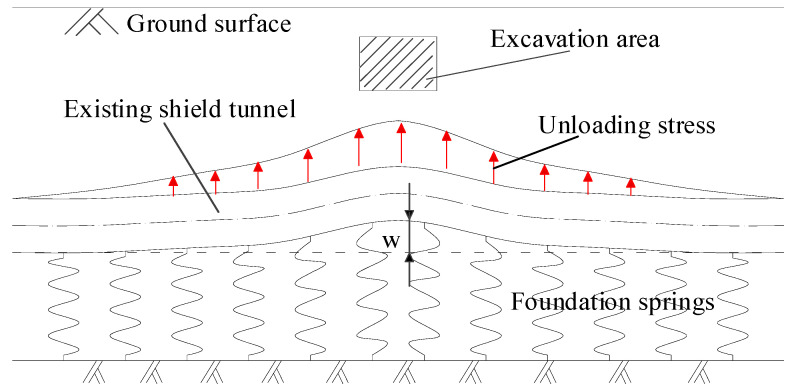
Single-sided elastic foundation beam model for shallow tunnel.

Figure 1 is the schematic diagram of force and deformation characteristics of the shallow tunnel. The vertical surrounding rock pressure can be equivalent to the weight of the strata. After the excavation of the upper soil, the geostress under the base of the excavation area is released, which causes movements of the surrounding soil and underlying tunnel. The strata from the upper part of the tunnel to the surface will experience follow-up deformation. In other words, the shield tunnel is only affected by foundation reaction forces from the lower strata. Therefore, the single-sided elastic foundation beam model is more suitable for shallow tunnels.

**Figure 2:**
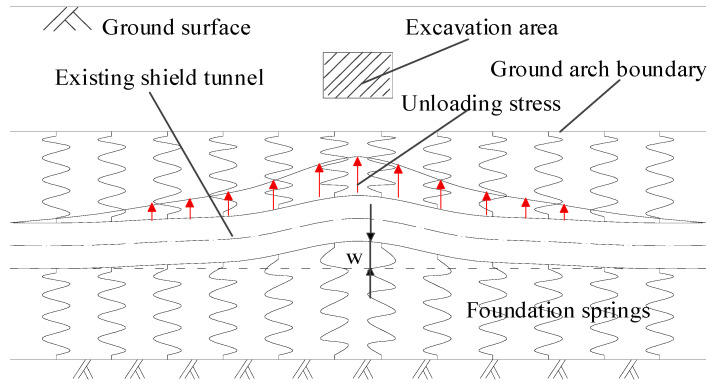
Double-sided elastic foundation beam model for deep tunnel.

Figure 2 shows the force and deformation characteristics of the deep tunnel. In the case of a deep tunnel, according to Protodyakonov’s pressure arch theory, the vertical earth pressure on the vault of the tunnel is smaller than the weight of the overlying strata due to the formation of a self-stable ground arch. It is commonly considered equivalent to the weight of the strata below the ground arch. The boundary of the ground arch provides reaction fulcrums for the upper foundation reaction forces and makes the deep tunnel bear double-sided foundation springs. This is the double-sided elastic foundation beam model of deep tunnels. Next, two situations must be discussed. In the first case, if the excavated soil is above the ground arch and the ground arch structure is not damaged, the existing tunnel will still be subjected to complete double-sided foundation springs during the above-crossing tunnelling. In the second case, if the excavated soil is located in the height range of the ground arch, the existing tunnel is only subjected to single-sided foundation reaction forces from the lower stratum in the excavation range and double-sided foundation reaction forces outside the excavation boundary. The model in the latter case can be called as partial missing double-sided elastic foundation beam model. This model can be used in the longitudinal deformation calculation of deep shield tunnels caused by the upper load reduction.

### 2.2. Force Analysis of Deep Shield Tunnel

**Figure 3:**
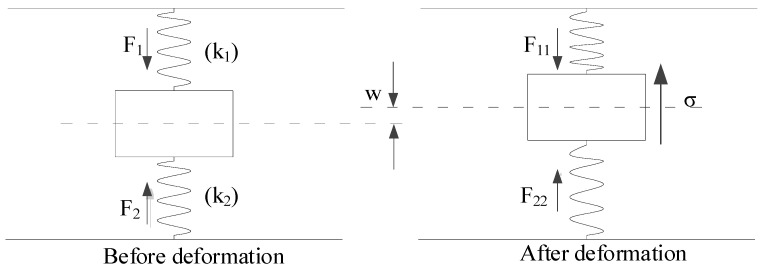
Force diagram of tunnel unit.

**Figure 4:**
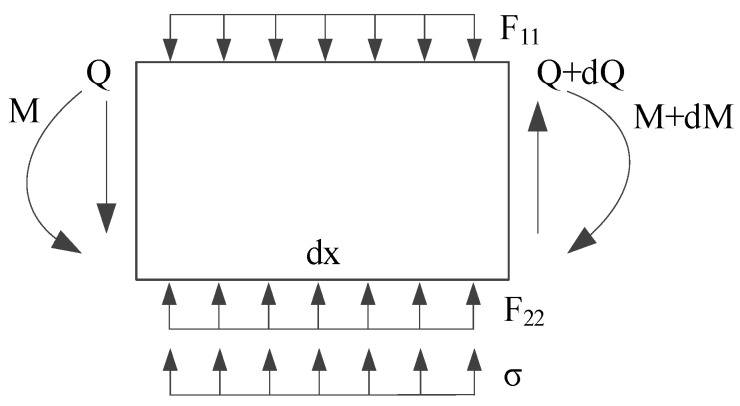
Force diagram of tunnel micro-unit.

The force diagram of the double-sided elastic foundation beam model of a deep shield tunnel is established in this study, as shown in Figure 3. First, the following assumptions are made: (1) the shield tunnel is in close contact with the surrounding soil, and there is no slip between them; (2) the stratum is a homogeneous isotropic elastomer; and (3) the foundation reaction forces satisfy the requirement of a linear elastic change in the deformation range of the shield tunnel. By dividing the shield tunnel into various tiny cells along the longitudinal direction, the forces of the tunnel microunit are shown in Figure 4. In the figures, $F_{1}$ and $F_{2}$ are the foundation reaction forces before the existing tunnel deformation; $F_{11}$ and $F_{22}$ are the foundation reaction forces after the existing tunnel deformation; $k_{1}$ and $k_{2}$ are the subgrade modulus coefficients of strata above and below the tunnel; σ is the vertical unloading stress on the existing shield tunnel; w is the vertical deformation of the existing shield tunnel; M is the bending moment; and Q is the shear force. If the excavated soil is under the ground arch, the foundation reaction force $F_{11}$ is zero within the excavated boundary.

Before the above-crossing construction, the shield tunnel is in equilibrium, and the relationship between the foundation reaction forces is as follows:

(1) $$F_{1}+mg=F_{2}$$

where mg is the gravity of the tunnel microunit.

After the above-crossing construction, the shield tunnel deforms, and the foundation reaction forces satisfy the following equation:

(2) $$\begin{array}{c} F_{11}{=F}_{1}+k_{1}w\\ F_{22}{=F}_{2}-k_{2}w \end{array}\}$$

Taking any tunnel microunit with width dx as the object of study, the following equation according to the static equilibrium condition in the vertical direction can be obtained as:

(3) $$\left(Q+dQ\right)-Q+F_{22}dx+\sigma dx-F_{11}dx-mg=0$$

From the moment equilibrium condition, the equation can be concluded as:

(4) $$M-\left(M+dM\right)+\left(Q+dQ\right)dx+F_{22}\frac{dx^{2}}{2}+\sigma \frac{dx^{2}}{2}-F_{11}\frac{dx^{2}}{2}-mg\frac{dx^{2}}{2}=0$$

In the mechanics of materials, the relationships of rotation angle θ, bending moment M and shear force Q of the beam are as follows:

(5) $$\begin{array}{c} \theta =\frac{dw}{dx}\\ M=-EI\frac{d\theta }{dx}=-EI\frac{d^{2}w}{dx^{2}}\\ Q=\frac{dM}{dx}=-EI\frac{d^{3}w}{dx^{3}} \end{array}\}$$

Omitting the second-order items in Equation (4), the deflection of the differential equation of the beam on a double-sided Winkler foundation from Equations (1)–(5) is obtained as:

(6) $$EI\frac{d^{4}w}{dx^{4}}+\left(k_{1}+k_{2}\right)w=\sigma$$

The preceding paragraphs show that if the shield tunnel is shallow, the upper strata do not provide foundation reaction forces. Then, the subgrade modulus coefficient $k_{1}$ is zero, and Equation (6) is consistent with the deflection differential equation of the single-sided elastic foundation beam model [10,11,12]. For the deep shield tunnel, two cases must be considered: (1) the excavated soil is above the ground arch. The forces on the shield tunnel conform to the double-sided Winkler foundation beam model, and the upward deformation of the existing tunnel can be calculated according to Equation (6). (2) The excavated soil is located in the ground arch height region. The shield tunnel is subjected to two-sided foundation reaction forces outside the excavated boundary and only single-sided foundation reaction forces within the excavated width. By introducing function f(x) with only two dependent variables 0 and 1, an equation that satisfies the partial missing two-sided elastic foundation beam model is established.

(7) $$EI\frac{d^{4}w}{dx^{4}}+\left[f\left(x\right)k_{1}+k_{2}\right]w=\sigma$$

(8) $$f\left(x\right)=\{\begin{array}{cc} 0, & -\frac{b}{2}\leq x\leq \frac{b}{2}\\ 1, & x<-\frac{b}{2},x>\frac{b}{2} \end{array}$$

where the axis of the excavation area is taken as the origin of the *x*-axis coordinate, and b is the width of the excavation area.

### 2.3. Calculation of Vertical Unloading Stress

**Figure 5:**
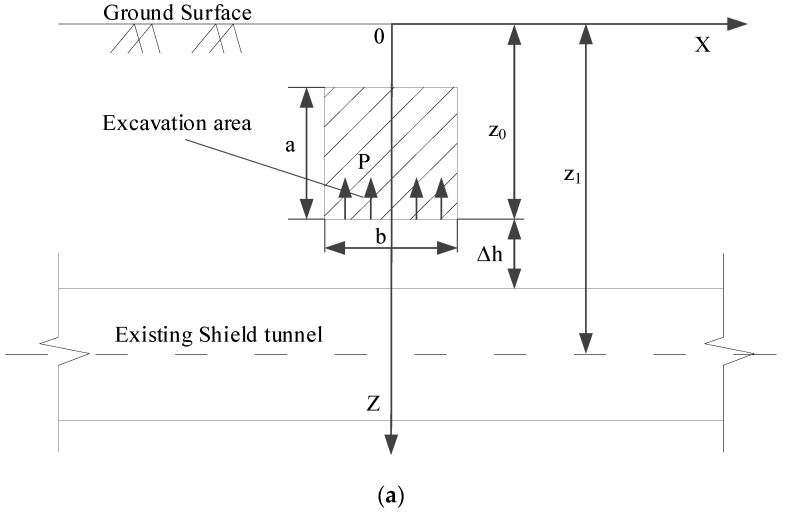

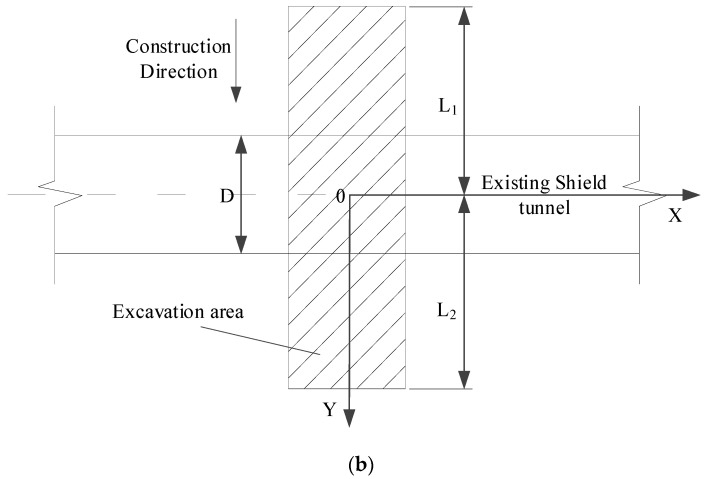
Calculation model diagrams: (**a**) Front view and (**b**) Bird’s-eye view.

Schematic diagrams of the calculation model associated with the above-crossing tunnelling are established, as shown in Figure 5.

After excavation, the upward unloading pressure P is generated on the floor of the excavated area. P is the difference between the weight of excavated soils and the support structure per unit excavation length. In Section 2.2, the authors assume that the stratum is a homogeneous isotropic elastomer. According to the principle of elastic mechanics, when a vertical concentrated force acts inside an elastic semi-infinite space, an arbitrary point in the semi-infinite space will be subjected to a vertical additional stress caused by this concentrated force. The magnitude of the additional stress was answered by Mindlin in 1936 [20]. Therefore, Mindlin’s solution is used in this paper. Without considering the effect of the existing shield tunnels on the additional stress, the vertical unloading stress σ(x) at an arbitrary point (x, 0, $z_{1}$) on the shield tunnel axis level, which is caused by upward unloading pressure P at an arbitrary point (ξ, η, $z_{0}$) on the floor of the excavation area, can be obtained.

(9) $$\sigma (x)=\int_{-L_{1}}^{L_{2}}\int_{-\frac{b}{2}}^{\frac{b}{2}}\frac{P\cdot d\xi \cdot d\eta }{8\pi \left(1-\mu \right)}\left[\begin{array}{l} -\frac{\left(1-2\mu \right)\left(z_{1}-z_{0}\right)}{R_{1}^{3}}+\frac{\left(1-2\mu \right)\left(z_{1}-z_{0}\right)}{R_{2}^{3}}-\frac{3{\left(z_{1}-z_{0}\right)}^{3}}{R_{1}^{5}}\\ -\frac{3\left(3-4\mu \right)z_{1}{\left(z_{1}+z_{0}\right)}^{2}-3z_{0}\left(z_{1}+z_{0}\right)\left(5z_{1}-z_{0}\right)}{R_{2}^{5}}\\ -\frac{30z_{0}z_{1}{\left(z_{1}+z_{0}\right)}^{3}}{R_{2}^{7}} \end{array}\right]$$

where $R_{1}=\sqrt{{\left(x-\xi \right)}^{2}+{\left(0-\eta \right)}^{2}+{\left(z_{0}-z_{1}\right)}^{2}}$ and $R_{2}=\sqrt{{\left(x-\xi \right)}^{2}+{\left(0-\eta \right)}^{2}+{\left(z_{0}+z_{1}\right)}^{2}}$ μ is the Poisson’s ratio; $z_{1}$ is the distance from the axis of the shield tunnel to the surface; $z_{0}$ is the distance from the floor of the excavation area to the surface; b is the width of the excavation area; $L_{1}$ is the distance from the start of the excavation area to the intersection of the axes; and $L_{2}$ is the distance from the intersection of the axes to the end of the excavation area.

### 2.4. Solution of Vertical Displacement

When the excavation area is under the ground arch, the displacements of the shield tunnel can be calculated by Equation (7). However, Equation (7) is a fourth-order partial differential equation with coefficient variables, and no analytical solution is available. Therefore, this paper uses the finite difference method to obtain the solution. First, the shield tunnel is discretized into N segments along the longitudinal direction. The length per segment is l, and N+1 nodes are generated. The deflections of the tunnel at the nodes are $\left(w_{0},w_{1},w_{2}\ldots w_{n-1},w_{n}\right)$, where $w_{0}$ and $w_{n}$ are the end nodes. Thus, Equation (7) can be approximately expressed by the finite difference equation as follows:

(10) $$EI\frac{1}{l^{4}}\left(w_{n-2}-4w_{n-1}+6w_{n}-4w_{n+1}+w_{n+2}\right)+f\left(x\right)k_{1}w_{n}+k_{2}w_{n}=\sigma$$

Since the shield tunnel is unaffected by tunnelling when the distance from the excavated area tends to infinity, both ends of the infinite beam are free ends, and the bending moments of nodes 0 and n are zero. According to the second-order difference equation, it can be obtained:

(11) $$\begin{array}{l} M_{0}=-EI\frac{d^{2}w}{dx^{2}}=-EI\frac{w_{0}-2w_{1}+w_{2}}{l^{2}}=0\\ M_{n}=-EI\frac{d^{2}w}{dx^{2}}=-EI\frac{w_{n-2}-2w_{n-1}+w_{n}}{l^{2}}=0 \end{array}\}$$

By solving Equation (11), the following equations can be obtained:

(12) $$\begin{array}{c} w_{0}=2w_{1}-w_{2}\\ w_{n}=2w_{n-1}-w_{n-2} \end{array}\}$$

Equation (10) can be written in matrix form, and the expression is:

(13) [A]{W}+[B]{W}+[C]{W}={σ}

where {W} is the elemental nodal vertical displacement vector; {σ} is the elemental nodal vertical unloading stress vector; [A] is the deformation stiffness matrix of the shield tunnel; [B] is the upper foundation spring stiffness matrix; and [C] is the lower foundation spring stiffness matrix. The abovementioned matrices and vectors are shown in Appendix A.

Equation (13) is rewritten as follows:

(14) ([A]+[B]+[C]){W}={σ}

Thus, the process of solving the fourth-order partial differential Equation (7) is transformed into solving a system of linear equations. The vertical displacements from node 1 to node (n−1) can be calculated by Equation (14), and the vertical displacements of both ends of the beam can be obtained by Equation (12).

## 3. Determination of Calculation Parameters

The deformation response of the underlying tunnel is affected by the interaction between strata and bearing capacity of the existing tunnel to resist deformation. Therefore, the determination of the related parameters should be given special attention, such as the bending stiffness EI, which reflects the capacity of the shield tunnel to resist deformation, and the subgrade modulus coefficient k, which determines the magnitude of foundation movement.

### 3.1. Equivalent Bending Stiffness of Shield Tunnel

The bending stiffness EI is the product of Young’s modulus E of tunnel segments and the moment of inertia I of the tunnel section. In reality, because there are segmental lining joints at intervals of 1.2 or 1.5 m in the longitudinal direction, the shield tunnel has significantly smaller overall bending stiffness than a continuous concrete tubular structure. Therefore, some scholars have proposed the concept of the longitudinal equivalent bending stiffness of shield tunnels. At present, Shiba’s equivalent model is commonly used, which is based on the stiffness of the circumferential lining joints [21,22]. However, Shiba’s model exaggerates the effect of the joints, and the calculated equivalent bending stiffness value is approximately 1/15 of that of a continuous tunnel lining structure, which is obviously smaller than the field test value. Liao et al. [23] modified Shiba’s model considering the integrity between joints and segment linings. Liao concluded that the longitudinal equivalent bending stiffness of a shield tunnel was approximately 1/5~1/7 of that of a continuous tunnel, which is verified and more similar to the field test. Therefore, the longitudinal equivalent bending stiffness $EI_{eq}$ in this paper can be calculated as follows:

(15) $$EI_{eq}=\frac{E_{c}}{5\sim 7}\frac{\pi \left(D^{4}-d^{4}\right)}{64}$$

where $E_{c}$ is the Young’s modulus of shield tunnel segments; D is the outer diameter of the shield tunnel; and d is the inner diameter of the shield tunnel.

### 3.2. Subgrade Modulus Coefficient

Determining the parameter of the subgrade modulus coefficient is complex and affected by the size and distribution of the foundation pressure, soil compressibility, buried depth, field test conditions and other factors. For deep shield tunnels, this paper estimates the subgrade modulus coefficient k from an empirical formula in the reference literature [24].

(16) $$k=\frac{1.3E_{s}}{B\left(1-\mu^{2}\right)}\sqrt[{12}]{\frac{E_{s}B}{EI}}$$

where $E_{s}$ is the Young’s modulus of soils; B is the width of the beam; B=D in this paper; and EI is equal to $EI_{eq}$.

## 4. Engineering Cases Validation

### 4.1. Interchange Passages of Beijing Daxing International Airport Express Above-Crossing Metro Line 10 Project

**Figure 6:**
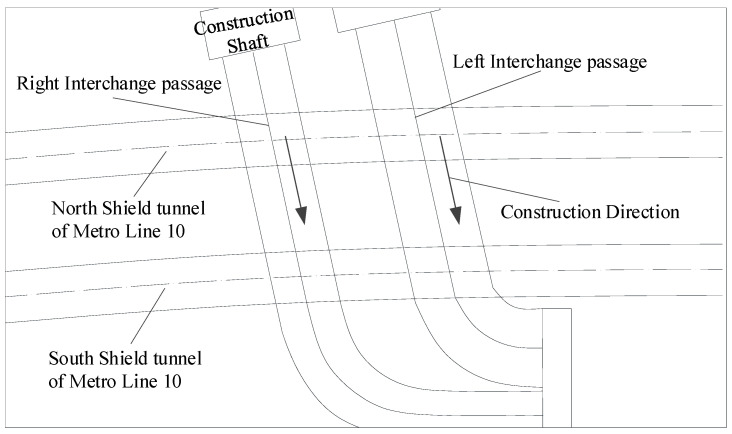
Layout of the interchange passages and Metro Line 10.

The underground interchange passages are set between the new Caoqiao station of Beijing Daxing International Airport Express and the existing Metro Line 10. The relative positions of the new passages and existing shield tunnels are shown in Figure 6. The interchange passages consist of two tunnels: the left line and the right line, which cross above the existing Metro Line 10 twin tunnels at an axial angle of 78°. During the above-crossing tunnelling, the left interchange passage will be first excavated until it crosses Metro Line 10; then, the construction of the right passage begins.

**Figure 7:**
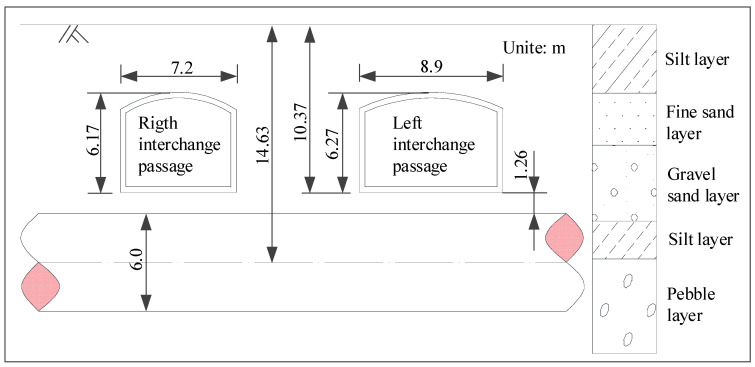
Sectional view of interchange passages and Metro Line 10.

Figure 7 shows the section form of the interchange passages and the stratum condition. Both interchange passages adopt the section form of the micro-arch and straight wall. The section of the left line is 8.9 m wide and 6.27 m high; the section of right line is 7.2 m wide and 6.17 m high; the buried depths of the bottom plate of both passages are 10.37 m. The existing shield tunnels have a circular cross-section with an outer diameter of 6.0 m, an inner diameter of 5.4 m and an axis depth of 14.63 m. The interchange passages are mainly located in the fine sand layer and gravel sand layer, which are moderately compressible and have a low water content. The existing Metro Line 10 is mainly in the pebble layer, which has high strength and low compressibility. The burial depth of the underground water level is 25 m, which is not considered in this paper.

**Figure 8:**
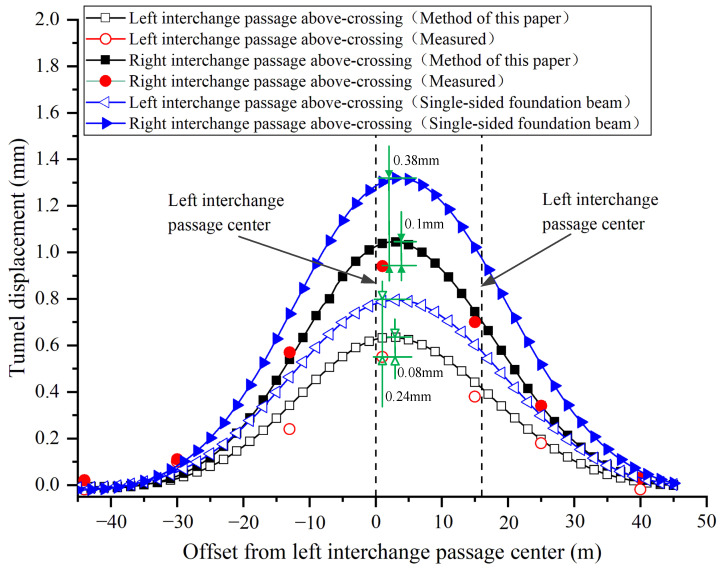
Comparison between the measured and calculated tunnel displacements due to the interchange passages above-crossing north shield tunnel of Line 10.

This paper takes the equivalent bending stiffness of the shield tunnel as 1/6 of the continuous tunnel. The model input parameters are shown in Table 1. Since the right interchange passage is only constructed after the left interchange passage above-crossing construction has been completed, the final displacements are obtained by the method of superposition of equal coordinate displacements caused by two passages of above-crossing tunnelling. The displacements caused by the construction shaft were subtracted from the measured values in the figure. Correspondingly, the effect of the shaft construction is not considered in the model calculation. The calculated and measured vertical displacements of the north shield tunnel of Metro Line 10 are shown in Figure 8.

As shown in Figure 8, the variation trends of the curves obtained by the proposed method and traditional model are similar to the measured distribution curves. Compared with the single-sided foundation beam model, the proposed model provides a smaller but closer prediction result. For the most concerned maximum displacement in engineering, the predicted value is basically consistent with the observed result. According to the mechanism analysis in Section 2.1, the underlying tunnel is subjected to two-sided foundation reaction forces outside the excavated boundary, so it bears greater uplift resistance. Due to the existence of shear stiffness between the segment rings, the tunnel uplift within the excavated width is weakened. Therefore, the proposed model is reasonable to predict the deformation of the existing shield tunnel.

### 4.2. Metro Line 8 Above-Crossing Existing Metro Line 2 Project in Shanghai

Chen et al. [4] reported a case where Metro Line 8 crossed above the existing Metro Line 2 in the interval between Qufu Road Station and People’s Square Station in Shanghai, China. Metro Line 8 consists of two parallel running tunnels, which are driven by the earth pressure balance shield with an excavated diameter of 6.34 m. The outer and inner diameters of Line 8 shield tunnels are 6.2 and 5.5 m, respectively, and the buried depths of axes of the shield-driven tunnels are 5.5 m. Metro Line 2 is composed of two directional tunnels: the North line and South line, whose outer and inner diameters are 6.2 and 5.5 m, and the buried depths of the axes are 13.1 m. The axial angle between Line 8 and Line 2 is 76°. The stratum environment of the project is mainly a silty and muddy clay layer, which is classified as a Quaternary soft clay layer. It is a typical Shanghai soft ground condition with low strength, high water content and high compressibility. Detailed engineering and geological conditions can be obtained in the reference.

The earliest study of this case is mainly deformation monitoring research, so there is no detailed physical parameterized introduction to the stratum. Therefore, this paper adopts the test results of Quaternary muddy clay, which is widely distributed over the Shanghai area, and obtains the undrained shear strength $C_{u}$ and Poisson’s ratio from reference [26]. According to the conclusion by Hashimoto et al. [27], the Young’s modulus $E_{s}$ of soft soil can be calculated by the Equation (17).

(17) $$E_{s}=350C_{u}$$

The model input parameters are shown in Table 2.

**Figure 9:**
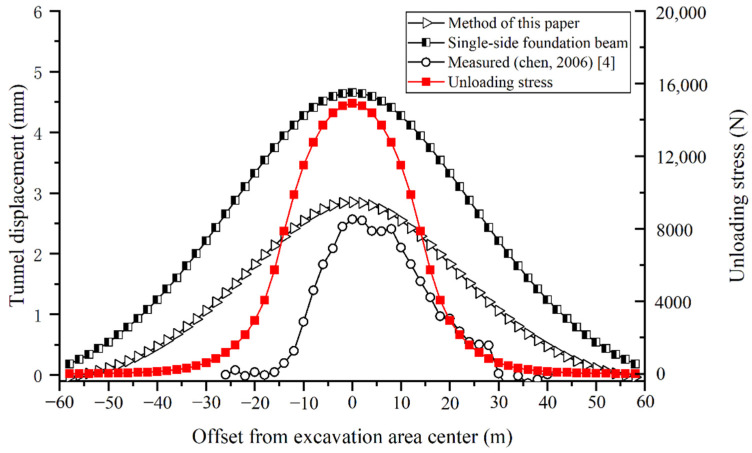
Comparison between the measured and calculated tunnel displacements due to the M8 above-crossing north shield tunnel of Line 2.

Figure 9 shows the comparison between the model calculation and the actual measurement. It can be found that the measured maximum tunnel displacement is 2.57 mm. The calculated maximum upheaval by the proposed model is 2.85 mm, which is 11% larger than the measured value. The calculated maximum upheaval by the single-side foundation beam model is 4.65 mm, which is 81% larger than the measured value. The proposed model predicts a smaller and closer deformation than the traditional model. The maximum tunnel displacement obtained by the method in this paper is basically consistent with the measured value. However, regardless of whether the method in this paper or the traditional method is used, the longitudinal influence range of the calculation is larger than the actual measurement, which is similar to the calculation conclusions of the literature [12,13]. The reason is that the elastic foundation beam models simplify the shield tunnel as a continuous beam for calculation, whose displacement changes are continuous. In reality, shield tunnel segment staggering will occur during the above-crossing construction, which weakens the deformation transfer. Therefore, the fluctuation of the measured displacement curve is similar to the computational unloading stress curve. Nonetheless, the maximum displacement is generally the most important prediction value in engineering practice, and the proposed model provides a more accurate prediction for deep shield tunnels.

## 5. Conclusions

This paper investigates the longitudinal deformation model of deep shield tunnels. The following conclusions can be drawn:

(1) A partial missing double-sided elastic foundation beam model is proposed to predict the uplift of deep shield tunnels due to upper load reduction. The model assumes the tunnel to be a Euler–Bernoulli beam and subjected to two-sided foundation reaction forces. According to the model, the authors derive a fourth-order partial differential equation with coefficient variables and provide the matrix formulas to solve the equation by the finite difference method. In contrast with the single-side foundation beam model, it is more suitable for calculating the deformation of deep shield tunnels.

(2) In comparison with the measured results of two engineering cases in different cities, the computational maximum displacements are basically consistent with the field tests, which are the results of most concern. The tunnel segment staggering will weaken the deformation transfer, so the longitudinal influence range of the calculation is larger than the actual measurement.

(3) Although the deep shield tunnel is only subjected to single-sided foundation forces within the excavated width like a shallow tunnel, the shear stiffness between the segment rings will weaken the tunnel uplift within the excavated width. Therefore, the traditional single-side foundation beam models obviously exaggerate the effect of excavation.

## Figures and Tables

**Table 1:** Model input parameters for case 1.

Parameter	Value
shield tunnel segment	$E_{c}=3.45\times 10^{4}\;\mathrm{MPa}$ [25]
longitudinal equivalent bending stiffness	$EI_{eq}=1.26\times 10^{5}\;{\mathrm{MNgm}}^{2}$
gravel sand layers (k_1_)	$E_{s}=60\mathrm{MPa},\mu =0.3$
pebble layers (k_2_)	$E_{s}=130\mathrm{MPa},\mu =0.22$

**Table 2:** Model input parameters for case 2.

Parameter	Value
shield tunnel segment	$E_{c}=3.45\times 10^{4}\;\mathrm{MPa}$ [25]
longitudinal equivalent bending stiffness	$EI_{eq}=1.59\times 10^{5}{\;\mathrm{MNgm}}^{2}$
Quaternary muddy clay (k_1_ = k_2_)	$C_{u}=25\;\mathrm{kPa},\mu =0.4$

## Data Availability

The data presented in this study are available on request from the corresponding author.

## Nomenclature

**Symbols and Greek Letters**	**Nomenclature**
$F_{1}$($F_{2}$)	the foundation reaction forces before the existing tunnel deformation
$F_{11}$($F_{22}$)	the foundation reaction forces after the existing tunnel deformation
$k_{1}$($k_{2}$)	the subgrade modulus coefficients of strata above and below the tunnel
σ	the vertical unloading stress on the existing shield tunnel
w	the vertical deformation of the existing shield tunnel
M	the bending moment
Q	the shear force
mg	the gravity of the tunnel microunit
b	the width of the excavation area
P	the upward unloading pressure
μ	the Poisson’s ratio
$z_{1}$	the distance from the axis of the shield tunnel to the surface
$z_{0}$	the distance from the floor of the excavation area to the surface
$L_{1}$	the distance from the start of the excavation area to the intersection of the axes
$L_{2}$	the distance from the intersection of the axes to the end of the excavation area
{W}	the elemental nodal vertical displacement vector
{σ}	the elemental nodal vertical unloading stress vector
[A]	the deformation stiffness matrix of the shield tunnel
[B]	the upper foundation spring stiffness matrix
[C]	the lower foundation spring stiffness matrix
E	the Young’s modulus
I	the moment of inertia of the tunnel section
$E_{c}$	the Young’s modulus of the shield tunnel segments
D	the outer diameter of the shield tunnel
d	the inner diameter of the shield tunnel
$E_{s}$	the Young’s modulus of soils
B	the width of the beam
$EI_{eq}$	the longitudinal equivalent bending stiffness
$C_{u}$	the undrained shear strength

## Appendix A

The expressions of matrices and vectors of Equation (13) are expressed as:

(A1) $$\left\{W\right\}={\left\{w_{1},w_{2}\ldots w_{n-2},w_{n-1}\right\}}^{T}$$

(A2) $$\left\{\sigma \right\}={\left\{\sigma_{1},\sigma_{2}\ldots \sigma_{n-2},\sigma_{n-1}\right\}}^{T}$$

(A3) $$\left[A\right]=\frac{EI}{l^{4}}{\left[\begin{array}{ccccccccc} 1 & -4 & 6 & -4 & 1 & & & & 0\\ 0 & 1 & -4 & 6 & -4 & 1 & & & \\ 1 & -4 & 6 & -4 & 1 & & & & \\ & 1 & -4 & 6 & -4 & 1 & & & \\ & & \ddots & \ddots & \ddots & \ddots & \ddots & & \\ & & & 1 & -4 & 6 & -4 & 1 & \\ & & & & 1 & -4 & 6 & -4 & 1\\ & & & 1 & -4 & 6 & -4 & 1 & 0\\ 0 & & & & 1 & -4 & 6 & -4 & 1 \end{array}\right]}_{\left(N-1\right)\left(N-1\right)}$$

(A4) $$\left[B\right]=f\left(x\right)k_{1}{\left[\begin{array}{ccccccccc} 1 & & & & & & & & \\ & 1 & & & & & & & \\ & & 1 & & & & & & \\ & & & 1 & & & & & \\ & & & & \ddots & & & & \\ & & & & & 1 & & & \\ & & & & & & 1 & & \\ & & & & & & & 1 & \\ & & & & & & & & 1 \end{array}\right]}_{\left(N-1\right)\left(N-1\right)}$$

(A5) $$\left[C\right]=k_{2}{\left[\begin{array}{ccccccccc} 1 & & & & & & & & \\ & 1 & & & & & & & \\ & & 1 & & & & & & \\ & & & 1 & & & & & \\ & & & & \ddots & & & & \\ & & & & & 1 & & & \\ & & & & & & 1 & & \\ & & & & & & & 1 & \\ & & & & & & & & 1 \end{array}\right]}_{\left(N-1\right)\left(N-1\right)}$$
